# Exploring the biological functional mechanism of the HMGB1/TLR4/MD-2 complex by surface plasmon resonance

**DOI:** 10.1186/s10020-018-0023-8

**Published:** 2018-05-10

**Authors:** Mingzhu He, Marco E. Bianchi, Tom R. Coleman, Kevin J. Tracey, Yousef Al-Abed

**Affiliations:** 1 Center for Molecular Innovation, The Feinstein Institute for Medical Research, 350 Community Drive, Manhasset, New York, 11030 USA; 2 Chromatin Dynamics Unit, Division of Genetics and Cell Biology, San Raffaele University and San Raffaele Scientific Institute IRCCS, Via Olgettina 58, 20132 Milan, Italy; 3 Center for Biomedical Science, and Center for Bioelectronic Medicine, The Feinstein Institute for Medical Research, 350 Community Drive, Manhasset, New York, 11030 USA

**Keywords:** HMGB1, TLR4/MD-2 complex, Surface plasmon resonance (SPR), Antagonist, TLR4-signaling

## Abstract

**Background:**

High Mobility Group Box 1 (HMGB1) was first identified as a nonhistone chromatin-binding protein that functions as a pro-inflammatory cytokine and a Damage-Associated Molecular Pattern molecule when released from necrotic cells or activated leukocytes. HMGB1 consists of two structurally similar HMG boxes that comprise the pro-inflammatory (B-box) and the anti-inflammatory (A-box) domains. Paradoxically, the A-box also contains the epitope for the well-characterized anti-HMGB1 monoclonal antibody “2G7”, which also potently inhibits HMGB1-mediated inflammation in a wide variety of in vivo models. The molecular mechanisms through which the A-box domain inhibits the inflammatory activity of HMGB1 and 2G7 exerts anti-inflammatory activity after binding the A-box domain have been a mystery. Recently, we demonstrated that: 1) the TLR4/MD-2 receptor is required for HMGB1-mediated cytokine production and 2) the HMGB1–TLR4/MD-2 interaction is controlled by the redox state of HMGB1 isoforms.

**Methods:**

We investigated the interactions of HMGB1 isoforms (redox state) or HMGB1 fragments (A- and B-box) with TLR4/MD-2 complex using Surface Plasmon Resonance (SPR) studies.

**Results:**

Our results demonstrate that: 1) intact HMGB1 binds to TLR4 via the A-box domain with high affinity but an appreciable dissociation rate; 2) intact HMGB1 binds to MD-2 via the B-box domain with low affinity but a very slow dissociation rate; and 3) HMGB1 A-box domain alone binds to TLR4 more stably than the intact protein and thereby antagonizes HMGB1 by blocking HMGB1 from interacting with the TLR4/MD-2 complex.

**Conclusions:**

These findings not only suggest a model whereby HMGB1 interacts with TLR4/MD-2 in a two-stage process but also explain how the A-box domain and 2G7 inhibit HMGB1.

**Electronic supplementary material:**

The online version of this article (10.1186/s10020-018-0023-8) contains supplementary material, which is available to authorized users.

## Background

As its name implies, High Mobility Group Box 1 (HMGB1) is a small protein that migrates rapidly on SDS-PAGE gels and was first identified as a nonhistone chromatin-binding protein that has important biological activities in human health and diseases (Kang et al. 2014; Andersson and Tracey 2011; VanPatten and Alabed 2017). HMGB1 resides in the nucleus of most eukaryotic cells, where it functions as a transcriptional regulator facilitating the binding of several regulatory protein complexes to DNA (Wang et al. 2007; Stros 2010). Upon cellular activation, injury or death, HMGB1 translocates to the cytoplasm, where it can activate autophagy by interacting with beclin-1 (Kang et al. 2010), and to the extracellular medium, where it acts as a prototypic Damage Associated Molecular Pattern (DAMP) molecule. This DAMP has cytokine, chemokine, and growth factor activities, orchestrating the inflammatory and immune responses (Bianchi et al. 2017). When extracellular HMGB1 is released passively from damaged necrotic cells or actively from immune and/or stressed cells, it promotes inflammatory responses by binding to key pattern recognition receptors such as the Receptor for Advanced Glycation Endproducts (RAGE) and Toll-like Receptors (TLRs) (Schmidt et al. 2000). Upon binding to its receptors, HMGB1 induces nuclear translocation of nuclear factor-κB (NF-κB), leading to secretion of pro-inflammatory cytokines including tumor necrosis factor-α (TNF-α), interleukin-6 (IL-6), and interleukin-1β (IL-1β) (Wu et al. 2016).

**Fig. 1:**
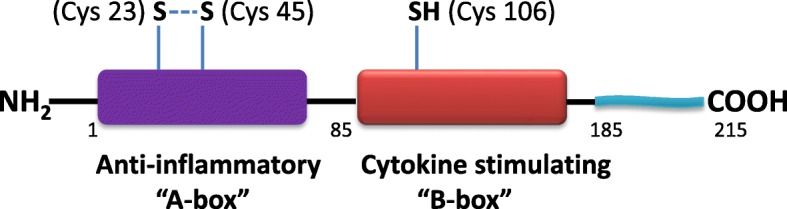
HMGB1 is composed of two box domains that individually have unique biological functions

HMGB1 is highly conserved among various species, with 99% identity between human, rat and bovine protein sequences (Sessa and Bianchi 2007). Structurally, HMGB1 consists of a single 215-amino acid polypeptide organized into two DNA-binding domains linked by a short basic hinge, and an acidic C-terminal tail (Stros 2010) (Fig. 1). Each of the DNA binding, L-shaped HMG-box domains, termed A-box and B-box, is approximately 80 amino acids long and about 43% identical to the other (Read et al. 1993; Weir et al. 1993). The C-terminal tail consists of 30 acidic residues (aspartates and glutamates) (Weir et al. 1993). HMGB1 has three conserved, redox sensitive cysteines (Fig. 1). Two of the cysteine residues are located in A-box: Cys23 and Cys45. These residues can rapidly form an intramolecular disulfide bond, and the redox reaction is reversible (Sahu et al. 2008). The formation of the disulfide bond in the A-box induces significant structural change in the loop, particularly, the flipping of Phe38 ring which is the key residue interacting with cisplatinated DNA (Wang et al. 2013). The third cysteine residue, Cys106, remains in its reduced state in B-box (Yang et al. 2013a). The oxidative state of the three cysteines determines the receptor preference of extracellular HMGB1 (Yang et al. 2013a).

HMGB1-TLR4 signaling has been strongly implicated in the pathogenesis of sterile injury (Maroso et al. 2010; Yang et al. 2013b). TLR4-deficient animals are significantly protected from tissue injury during hepatic ischemia. TLR4, a pivotal receptor for activation of innate immunity, including cytokine release and tissue damage, is required for HMGB1-dependent activation of macrophage TNF release, whereas RAGE and TLR2 are dispensable. TLR4 activity and interaction with its ligands depend on a molecular collaboration with the extracellular adaptor protein Myeloid Differentiation factor 2 (MD-2) (Miyake 2004; Visintin et al. 2006). Surface plasmon resonance (SPR) studies indicate that HMGB1 binds specifically to TLR4/MD-2, and that this binding requires cysteine 106 (Yang et al. 2010). Recently, we demonstrated that MD-2 binds specifically to the disulfide isoform of HMGB1 and not to the other isoforms (Yang et al. 2015a).

Structure–function analyses demonstrated that exogenous B-box recapitulates the cytokine activity of full length HMGB1 and stimulates TNF-훼 release from macrophages. TNF-stimulating activity localizes to 20 amino acids within B-box (B1-B20, HMGB1 amino acids 89 to 108) (Li et al. 2003). In contrast, despite a 40% sequence identity with B-box, A-box not only possesses no TNF-stimulating activity but also acts as an antagonist of HMGB1, and can compete with HMGB1 for binding sites on the surface of activated macrophages and suppresses HMGB1-induced pro-inflammatory cytokines release (Yang et al. 2004). Recombinant A-box was found protective in established preclinical inflammatory disease models (Andersson and Tracey 2011; Venereau et al. 2016), including mouse models of sepsis (Yang et al. 2004; Suda et al. 2006), lung injury induced by LPS, hepatitis, severe acute pancreatitis, ischemia-reperfusion injury in the heart, cerebral ischemia, and epilepsy (Maroso et al. 2010; Andrassy et al. 2008; Muhammad et al. 2008; Yuan et al. 2009). The specific mechanism of how A-box antagonizes HMGB1 remains unknown. Adding to this mystery is the fact that the A-box contains the epitope of the anti-HMGB1 monoclonal antibody, 2G7. How 2G7 inhibits HMGB1 activity through binding to the A-box remains unknown.

As it has long been known that A-box acts as a potent HMGB1 antagonist in many experimental models, we sought to address the underlying molecular mechanism using surface plasmon resonance (SPR). We found that A-box binds to TLR4 with a comparable equilibrium dissociation constant (K_D_) to HMGB1, but with a 10-fold slower dissociation rate than that of HMGB1. We propose that A-box antagonizes HMGB1 by blocking HMGB1-TLR4 binding sites, thus preventing HMGB1/TLR4/MD-2 complex formation.

## Methods

### Reagents

Human TLR4/MD-2 complex, human MD-2, TLR4 were obtained from R&D Systems.

Recombinant HMGB1 was expressed in *E. coli* and purified to homogeneity as described previously (Wang et al. 1999; Li et al. 2004). DTT reduced HMGB1 was prepared as previously described (Yang et al. 2012). The non-oxidizable HMGB1 3S mutant, where serines replace cysteines, was provided by HMGBiotech (Milan, Italy). Recombinant GST-A-box, GST-B-box and GST protein were expressed in *E. coli* and purified to homogeneity as previously described (Li et al. 2003; Yang et al. 2004; Li et al. 2004). Recombinant A-box without GST tag was also provided by HMGBiotech (Milan, Italy). A-box with or without GST tag showed comparable binding activity in our SPR assay (Additional file 1: Figure S4), both types of A-box were utilized in this study. Typically, LPS content in the protein preparations was less than 1 pg LPS/μg protein.

### Surface plasmon resonance analysis

Biacore T200 (GE Healthcare, USA) was used for real-time binding interaction studies. Binding reactions were done in HBS-EP buffer from BIAcore, containing 10 mM hepes, 150 mM NaCl, 3 mM EDTA and 0.05% surfactant p20, pH 7.4. At least 3 independent experiments were performed.

### TLR4/MD-2 or TLR4 and redox forms of HMGB1 binding analyses

A slow, high-level immobilization of TLR4/MD-2 or rhTLR4 protein was obtained on a CM5 series chip (GE Healthcare). The TLR4/MD-2 complex was diluted to a concentration of 20 μg/mL in 10 mM Acetate buffer (pH 4.5). A 1:1 mixture of N-hydroxysuccinimide and N-ethyl-N-(dimethyaminopropyl)carbodiimide was used to activate 2 flow-cells of the CM5 chip. One flow-cell was used as a reference and thus immediately blocked upon activation with 1 M ethanolamine (pH = 8.5). The sample flow-cell was injected with the diluted TLR4/MD-2 at a flow rate of 10 μL/min. The TLR4/MD-2 injection was stopped when the surface plasmon resonance reached ~ 1200 RU. For TLR4/MD-2 and HMGB1 isoform kinetics assays, HMGB1 isoforms were sequentially injected at a flow rate of 30 μL/min for 60s at 25 °C; the dissociation time was set for 1 min. The concentrations were 31.25, 62.5, 125, 250, 500, 1000 nM for disulfide and reduced HMGB1; 310, 625, 1250, 2500, 5000 nM for 3S mutant HMGB1. The rhTLR4 protein was diluted to a concentration of 20 μg/mL in 10 mM Acetate buffer (pH 5.0) and was immobilized on the CM5 chip according to the same amine coupling method described above. The TLR4 injection was stopped when the surface plasmon resonance reached ~ 800 RU. For TLR4 and HMGB1 isoform kinetics assays, HMGB1 isoforms were sequentially injected at a flow rate of 30 μL/min for 60s at 25 °C, the dissociation time was set for 1–2 min. The concentrations were 31.25, 62.5, 125, 250, 500, 1000 nM for disulfide and reduced HMGB1; 250, 500, 1000, 2000, 4000, 8000 nM for 3S mutant HMGB1. The equilibrium dissociation constant (K_D_) for individual analytes was obtained to evaluate the binding affinity by using the BIAEvaluation 2.0 software (GE Healthcare) supposing a 1:1 binding ratio.

### TLR4/MD-2 and GST-A-box or GST-B-box binding analyses

TLR4/MD-2 protein was immobilized onto a CM5 series chip (GE Healthcare) by amine coupling chemistry. GST protein alone was used as negative control (Additional file 1: Figures S2-S3). GST-A-box or GST-B-box were sequentially injected over immobilized TLR4/MD-2 (1100 RU) at a flow rate of 30 μL/min for 60s at 25 °C; the dissociation time was set for 1 min. The concentrations were 150, 310, 625, 1250, 2500, 5000 nM for GST-A-box and GST-B-box. The association and dissociation phases of GST-A-box were separately fitted to a 1:1 L binding model provided in the BIAevaluation 2.0 software. Binding affinity of GST-B-box was determined by global fitting of data to a steady-state affinity model in the BIAEvaluation 2.0.

### TLR4 and GST-A-box or GST-B-box binding analyses

TLR4 protein was immobilized onto a CM5 series chip (GE Healthcare) by amine coupling chemistry. GST-A-box was sequentially injected over the immobilized TLR4 (800 RU) at a flow rate of 30 μL/min for 60s at 25 °C; the dissociation time was set for 2 min. The concentrations were 310, 625, 1250, 2500, 5000, 10,000 nM. The equilibrium dissociation constant (K_D_) was obtained by using the BIAEvaluation 2.0 software (GE Healthcare) supposing a 1:1 binding ratio. 1 μM and 5 μM GST-B-box was injected over the immobilized TLR4 at a flow rate of 30 μL/min for 60s at 25 °C, however there has no observed binding activity. GST protein alone was used as negative control; no binding was observed (Additional file 1: Figures S2-S3).

### MD-2 and GST-A-box or GST-B-box binding analyses

GST-A-box or B-box protein was immobilized onto a CM5 series chip (GE Healthcare) by amine coupling chemistry. MD-2 was sequentially injected over the immobilized GST-A-box (750 RU) or GST-B-box (1000 RU) at a flow rate of 30 μL/min for 60s at 25 °C; the dissociation time was set for 1 min. The concentrations were 17, 31.25, 62.5, 125, 250, 500 nM. Apparent equilibrium dissociation constants (K_D_) were determined by global fitting of data to a steady-state affinity model in the BIAEvaluation 2.0 software. Binding affinity and kinetics data of HMGB1 isoforms and segments to TLR4/MD-2 receptors are listed in Additional file 1: Tables S1-2S. Data shown in this report are representative of three independent experiments.

### Biacore analysis of complex formation among TLR4/MD-2, TLR4, A-box and HMGB1

The assay was performed using the HBS-EP buffer from BIAcore as described above. HMGB1 (100 nM) was injected on a TLR4/MD-2 complex sensor chip surface followed by HBS buffer or HMGB1 (100 nM) or TLR4/MD-2 (100 nM) or mixture of two proteins (1:1 M ratio) using the dual injection command. In competition experiments, A-box (10 μM) was injected on a TLR4 sensor chip surface followed by 2-fold dilutions of HMGB1 (2.5–5 μg/ml) using the dual injection command. In separate experiments, HMGB1 (500 nM) was injected on a TLR4 sensor chip surface, followed by A-box using the dual injection command (Additional file 1: Figure S5).

## Results

### The TLR4/MD-2 complex is sensitive to the oxidative state of HMGB1, while TLR4 is not

**Fig. 2:**
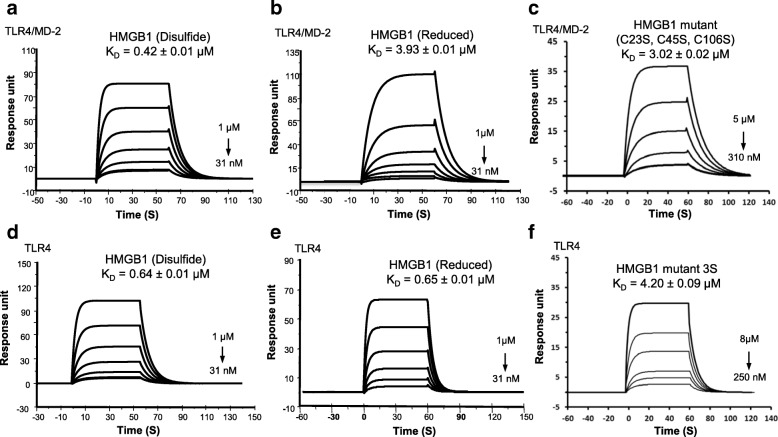
SPR analyses of redox forms of HMGB1 binding to TLR4/MD-2 complex or TLR4. **a**-**c** TLR4/MD-2 complex was coated on the CM5 chip; disulfide HMGB1 binds to complex with a K_D_ of 0.42 ± 0.01 μM; reduced HMGB1 binds with a K_D_ of 3.93 ± 0.01 μM; HMGB1 3S mutant binds with a K_D_ of 3.02 ± 0.02 μM. **d**-**f** TLR4 was coated on the chip; HMGB1 binds to TLR4 with a K_D_ of 0.64 ± 0.01 μM; reduced HMGB1 binds with a K_D_ of 0.65 ± 0.01 μM; HMGB1 3S mutant binds with a K_D_ of 4.20 ± 0.09 μM. Data are representative of three repeats

Recent studies emphasize that the redox states of the three conserved cysteine residues within HMGB1 regulate its receptor-binding ability and subsequent biological outcome including its pro-inflammatory activity (Yang et al. 2013a). In 2015, we demonstrated that MD-2 binds specifically to the cytokine-inducing disulfide isoform of HMGB1, whereas fully reduced or sulfonyl HMGB1 had 1000-fold lower binding affinity for MD-2 (Yang et al. 2015b). To further clarify the underlying molecular mechanisms, we examined whether the TLR4/MD-2 complex or TLR4 alone could discriminate various HMGB1 isoforms. We tested reduced HMGB1 and the non-oxidizable HMGB1 3S mutant, generated by replacing all three cysteines with serines. To begin, the TLR4/MD-2 complex was coated on the CM5 sensor chip, and then probed with each of the three HMGB1 isoforms (Fig. 2a-c). Consistent with previous findings (Yang et al. 2012), the disulfide HMGB1 isoform binds to TLR4/MD-2 in a concentration-dependent manner, with relatively high affinity, an apparent equilibrium dissociation constant (K_D_) of 0.42 ± 0.01 μM (Fig. 2a). In contrast, DTT-reduced HMGB1 and the non-oxidizable 3S mutant bind TLR4/MD-2 complex with ~ 10-fold lower affinity (apparent K_D_ = 3.93 ± 0.01 and 3.02 ± 0.02 μM, respectively, Fig. 2b & c). These results are consistent with the finding that reduced HMGB1 and 3S do not induce cytokine expression in macrophages (Yang et al. 2013a; Venereau et al. 2012). The sensorgrams also demonstrate that the disulfide HMGB1 has a 10-fold faster association rate (ka = 2.86 ± 0.03 × 10^5^ M^− 1^ s^− 1^, Additional file 1: Table S2) relative to the other isoforms.

Previously, we reported that HMGB1 was incapable of directly binding to TLR4 in the absence of MD-2 (Yang et al. 2015a). In this study, we revisited this observation and refined the experimental conditions (immobilization levels and/or switching ligand and analyte) for this binding assay. Using these optimized conditions we found that disulfide HMGB1 does bind to TLR4 (coated on the sensor chip) in a concentration-dependent manner with an apparent K_D_ of 0.64 ± 0.01 μM (Fig. 2d). Next, the other isoforms of HMGB1 were injected onto the TLR4 sensor chip to investigate which redox state of HMGB1 was most conducive to binding (Fig. 2e-f). Reduced HMGB1 bound to TLR4 with a comparable equilibrium dissociation constant K_D_ = 0.65 ± 0.01 μM (Fig. 2e), indicating that a disulfide bond between C23 and C45 has a negligible influence on the binding to TLR4. However, the sensorgrams demonstrated a 2-fold slower dissociation rate (kd = 0.128 ± 0.002 s^− 1^, Additional file 1: Table S2) for disulfide HMGB1 compared with reduced HMGB1, indicating that the complex with TLR4 is more stable once formed. We also found that 3S, where C23, C45 and C106 are mutated to serines, binds to TLR4 with a weaker affinity (apparent K_D_ = 4.20 ± 0.09 μM) (Fig. 2f), suggesting that three cysteine residues within HMGB1 are essential for binding to TLR4.

### A-box binds to TLR4/MD-2 complex and major binding sites are located on TLR4

**Fig. 3:**
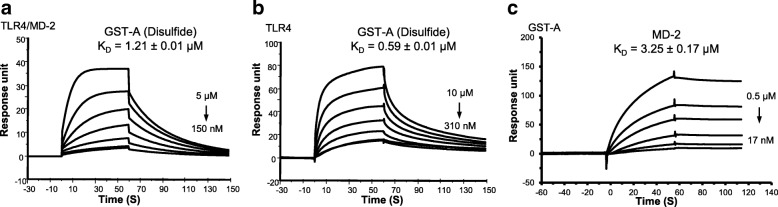
SPR analyses of GST-A-box binding to TLR4/MD-2, TLR4 and MD-2. **a** TLR4/MD-2 complex was coated on the CM5 chip, GST-A-box binds to complex with a K_D_ of 1.21 ± 0.02 μM. **b** TLR4 was coated on the chip, GST-A-box binds to TLR4 with a K_D_ of 0.59 ± 0.01 μM. **c** GST-A-box was coated on the CM5 chips, MD-2 was used as analyte, MD-2 binds to GST-A-box with a K_D_ of 3.25 ± 0.17 μM. GST tag was used as negative control. Data are representative of three repeats

As mentioned above, A-box has been widely used as an HMGB1 antagonist in many inflammatory disease models. Exogenous A-box has been reported to antagonize full-length HMGB1 by competitively binding to RAGE (Zhang et al. 2008); it was also described to interact with CXCL12 and thus competes with the HMGB1 for signaling via CXCR4 (Schiraldi et al. 2012). In the present study, we investigated the A-box–Toll-like receptor 4 (TLR4) interaction using SPR. TLR4/MD-2, TLR4 or MD-2 were individually immobilized on the CM5 sensor chip; recombinant GST-A-box was passed through the chips to analyze the binding activity. We observed that GST-A-box had a high affinity to TLR4 with apparent K_D_ of 0.59 ± 0.01 μM, compared to the TLR4/MD-2 (K_D_ = 1.21 ± 0.02 μM) (Fig. 3a-b). In addition, the sensorgrams showed that GST-A-box interacted with TLR4/MD-2 and TLR4 in comparable association, but different dissociation rate constants (Additional file 1: Tables S1-S2). For example, GST-A-box associated to TLR4/MD-2 at a rate of 3.08 ± 0.85 × 10^4^ M^− 1^ s^− 1^ and dissociated at 0.037 ± 0.01 s^− 1^; while associated to TLR4 at 2.20 ± 0.02 × 10^4^ M^− 1^ s^− 1^ and dissociated at a 3-fold slower rate of 0.013 ± 0.01 s^− 1^. On the contrary, only weak binding activity was observed on the MD-2 immobilized chip (Additional file 1: Figure S1). Therefore, we asked whether GST-A-box binds to MD-2 in the reverse orientation. We immobilized GST-A-box on the CM5 chip; MD-2 was injected as analyte. The sensorgram showed that MD-2 had a weak association rate with GST-A-box and the affinity of GST-A-box binding to MD-2 (K_D_ = 3.25 ± 0.17 μM) was much lower than binding to TLR4 or TLR4/MD-2 (Fig. 3c, Additional file 1: Tables S1-S2). Taken together, these results confirmed that A-box alone binds to the TLR4/MD-2 complex and major binding site(s) are likely located on TLR4.

### B-box binds to MD-2 but not TLR4

**Fig. 4:**
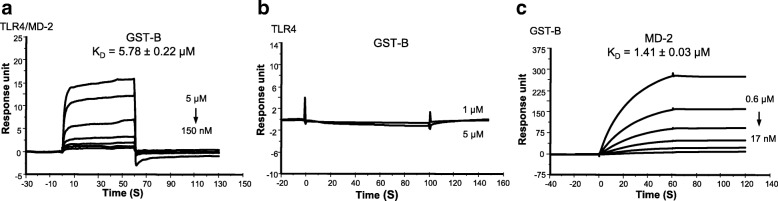
SPR analyses of GST-B-box binding to TLR4/MD-2, TLR4 and MD-2. **a** TLR4/MD-2 complex was coated on the CM5 chip, GST-B-box binds to TLR4/MD-2 complex with a K_D_ of 5.78 ± 0.22 μM. **b** TLR4 was coated on the chip, GST-B-box has no significant binding to TLR4. **c** GST-B-box was coated on the CM5 chips, MD-2 was used as analyte, MD-2 showed better binding affinity to GST-B-box (K_D_ of 1.41 ± 0.03 μM) relative to GST-A-box (K_D_ = 3.25 ± 0.17 μM). GST tag was used as negative control. Data are representative of three repeats

To evaluate the binding of B-box to the TLR4/MD-2 complex in detail, SPR analyses were performed. Akin to previous reports (Yang et al. 2010), the GST-B-box binds to TLR4/MD-2 in a concentration-dependent manner, with an apparent equilibrium dissociation constant K_D_ of 5.78 ± 0.22 μM (Fig. 4a), 6-fold weaker than GST-A-box (Fig. 3a). The sensorgram showed that GST-B-box has a fast association to TLR4/MD-2 complex and is quickly dissociated by washing, suggesting that the complex between GST-B-box and TLR4/MD-2 is not remarkably stable. Surprisingly, when GST-B-box was applied to immobilized TLR4, no significant binding was observed (Fig. 4b), and this lack of interaction was confirmed in the reverse orientation experiment (not shown). When GST-B-box was injected over immobilized MD-2, no binding activity was found (Additional file 1: Figure S1); in contrast, MD-2 showed a concentration-dependent binding to immobilized GST-B-box with a K_D_ of 1.41 ± 0.03 μM (Fig. 4c). The extremely slow off-rate indicated that the complex between GST-B-box and MD-2, once formed, was much more stable than the complex formed by GST-A-box. These data indicate that B-box alone binds to MD-2, but not to TLR4, suggesting that the binding of B-box to the TLR4/MD-2 complex occurs via the MD-2 subunit.

### Plausible mechanism of HMGB1-TLR4 signaling and the role of A-box as antagonist

Binding of HMGB1 to TLR4 has been shown to activate the MyD88 signaling pathway, thus resulting in the release of pro-inflammatory cytokines (Ugrinova and Pasheva 2017). It is unknown whether HMGB1 recognition by the TLR4/MD-2 complex shares similarities with other prototypical activators of TLR4 signaling, such as lipopolysaccharide (LPS). In the last decade, the crystal structures of the LPS receptors and accessory proteins have been characterized. Recently, Kim and coworkers reconstituted the entire cascade of LPS transfer to TLR4/MD-2 in a total internal reflection fluorescence (TIRF) microscope in a single-molecule analysis, reveling that a single LPS molecule bound to CD14 is transferred to TLR4/MD-2 in a TLR4-dependent manner (Kim and Kim 2017). Upon binding of LPS to a hydrophobic pocket in MD-2, two LPS-bound TLR4/MD-2 complexes form an M-shaped dimer, followed by activation of the signaling pathway (Kim and Kim 2017).

We hypothesized that HMGB1 may act in a manner similar to LPS, binding to TLR4/MD-2 complex and then inducing the dimerization of HMGB1/TLR4/MD-2 complexes, which brings together the cytosolic toll/interleukin-1 receptor (TIR) domains of TLR4 to recruit downstream adaptor molecules. To examine the details of the binding of HMGB1 to the TLR4/MD-2 complex, we immobilized one ligand on a Biacore CM5 sensor chip and used a sequential dual-injection method where the second analyte is injected after the first analyte with zero dissociation time. If a two-step increase in the bound mass is observed, we infer that the second analyte can bind to the complex of the ligand and first analyte; conversely, a decrease or unchanged signal after the second injection suggests that the first and second analytes compete for binding to the ligand, and then a ternary complex is not formed.

**Fig. 5:**
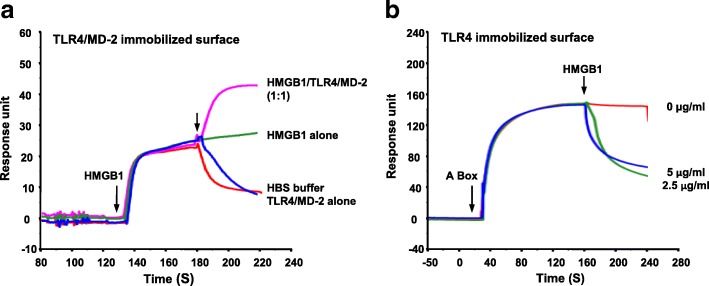
Ternary complex formation among TLR4/MD-2, TLR4, A-box and HMGB1. **a** Biacore sensorgram showing molecular interactions, after injecting HMGB1 (100 nM) followed immediately by HBS buffer, HMGB1 (100 nM) or TLR4/MD-2 (100 nM) or mixture of the latter (100 nM each) onto a TLR4/MD-2 sensor chip surface. **b** Injecting A-box (10 μM), followed immediately by A-box (0 μg/ml of HMGB1) or HMGB1 (2.5 and 5 μg/ml) onto a TLR4 sensor chip surface

HMGB1 (100 nM) was applied to the TLR4/MD-2 sensor chip surface and reached a steady state signal of ~30 RU (Fig. 5a). When blank control (HBS buffer) was injected as second analyte, the binding signal was decreased due to the dissociation of bound HMGB1. When a second injection of TLR4/MD-2 (100 nM) was made, no additional binding was observed. When HMGB1 (100 nM) was injected as second analyte, the binding signal was unchanged. Instead, when a mixture of HMGB1 and TLR4/MD-2 (1:1 M ratio) was injected, we observed a significant increase of the binding signal immediately after injection. These data suggest that HMGB1 induces dimerization between TLR4/MD-2 complexes, which could be the key step in the HMGB1-TLR4 signaling pathway. We next considered whether, as an HMGB1 antagonist, A-box exerts its role through interference with the formation of the HMGB1/TLR4/MD-2 complex. We immobilized TLR4 on the CM5 sensor chip and injected 10 μM A-box to reach a steady state binding of ~ 150 RU; after adding HMGB1 as second analyte, no complex with HMGB1 was seen (Fig. 5b). Similarly, we observed a decrease of the binding signal when we injected HMGB1 followed by A-box (data not shown, Additional file 1: Figure S5). These data demonstrated that A-box directly competes with HMGB1 for binding to TLR4.

## Discussion

**Scheme 1:**
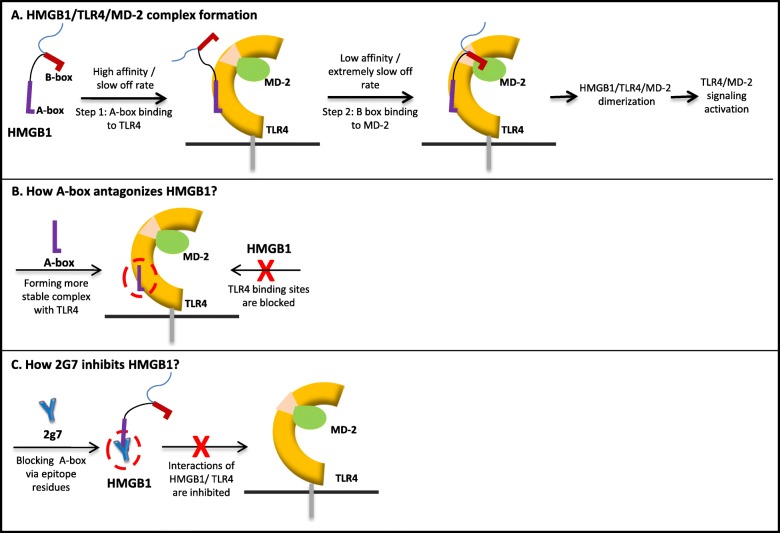
Proposed mechanism of HMGB1-TLR4 interaction and role of anti-HMGB1 antibody (2G7)

We propose the mechanism of HMGB1-induced TLR4 signaling outlined in Scheme 1. Real-time SPR studies showed that: 1) the binding of HMGB1 to TLR4/MD-2 complex is mostly contributed by the A-box domain whose major binding site(s) are located on TLR4; 2) B-box binds to MD-2; and 3) the HMGB1/TLR4/MD-2 complex can dimerize with another HMGB1/TLR4/MD-2 complex. Our results suggest that HMGB1/TLR4/MD-2 interaction is initiated by HMGB1-TLR4 binding via the A-box domain (high affinity and slow off-rate, Scheme 1a) and, once in close proximity, the HMGB1 B-box domain binds to MD-2 (low affinity but extremely slow off-rate, Scheme 1a). In addition, SPR studies also suggest that A-box functions as an HMGB1 antagonist by blocking the first step of HMGB1 binding to TLR4 (Scheme 1b). A-box and HMGB1 bind to TLR4 with comparable equilibrium dissociation constants (K_D_), but with quite different dissociation rates (Additional file 1: Table S2), indicating that the complex formed between A-box and TLR4 is more stable than that formed by HMGB1. As hypothesized in the Scheme 1b, when A-box and HMGB1 are present, A-box occupies and blocks HMGB1/TLR4 binding sites, preventing downstream full-length HMGB1/TLR4/MD-2 interactions, thus inhibiting TLR4/MD-2 signal activation.

Previously, SPR studies reported by Yang et al. (Yang et al. 2010) showed that anti-HMGB1 monoclonal antibody (mAb) 2G7 also inhibited the binding of HMGB1 to TLR4/MD-2. This antibody has been extensively used by researchers to block HMGB1 activity both in vitro and in vivo (Venereau et al. 2016; Ugrinova and Pasheva 2017). The epitope in HMGB1 that binds to 2G7 has been identified within amino acids 53–63 of the A-box subunit (Qin et al. 2006). However, the molecular mechanisms whereby binding of 2G7 to A-box has anti-inflammatory activity remained elusive. Based upon the finding of A-box as an HMGB1 antagonist, we speculate that mAb 2G7 would share similar mechanism to inhibit HMGB1 activity (Scheme 1c) by blocking HMGB1-TLR4 interactions.

## Conclusions

In summary, SPR studies showed that HMGB1 and its fragments (A-box and B-box) individually interact with the TLR4/MD-2 receptor with different binding and kinetic parameters. Our study also reveals that HMGB1 likely activates TLR4 signaling through inducing TLR4/MD-2 dimerization. The HMGB1 recognition cascade can be disrupted by the antagonistic A-box fragment due to its higher binding affinity to TLR4 or by blocking A-box with anti-HMGB1 mAb 2G7. Ongoing studies further detailing HMGB1/TLR4/MD-2 interactions will facilitate the design and development of therapeutics to inhibit HMGB1-mediated inflammation.

## Additional file

Additional file 1: **Table S1.** Binding affinity of HMGB1 isoforms and segments. **Table S2.** Kinetics data of HMGB1 isoforms and segments. **Figure S1.** SPR analyses of GST-A-box and GST-B box binding to MD-2. **Figure S2.** SPR analyses of GST-A-box binding to TLR4/MD-2, TLR4. **Figure S3.** SPR analyses of GST-B-box binding to TLR4/MD-2, TLR4. **Figure S4.** SPR analyses of GST-A-box and A-box binding to TLR4/MD-2, TLR4. **Figure S5.** Ternary complex formation among TLR4, A-box and HMGB1. (DOCX 241 kb)

## Abbreviations

DAMP: Damage Associated Molecular Pattern
DNA: Deoxyribonucleic acid
HMGB1: High Mobility Group Box 1
IL-1β: Interleukin-1β
IL-6: Interleukin-6
K_D_: Equilibrium dissociation constant
LPS: Lipopolysaccharide
MD-2: MYELOID Differentiation factor 2
NF-kB: Nuclear factor-κB
RAGE: Receptor for Advanced Glycation Endproducts
SDS-PAGE: Sodium dodecyl sulfate –polyacrylamide gel electrophoresis
SPR: Surface plasmon resonance
TIR: Toll/interleukin-1 receptor
TIRF: Total internal reflection fluorescence
TLRs: Toll-like Receptors (TLRs)
TNF-α: Tumor necrosis factor
