# Correction to: Exploring the biological functional mechanism of the HMGB1/TLR4/MD-2 complex by surface plasmon resonance

**DOI:** 10.1186/s10020-018-0030-9

**Published:** 2018-06-13

**Authors:** Mingzhu He, Marco E. Bianchi, Tom R. Coleman, Kevin J. Tracey, Yousef Al-Abed

**Affiliations:** 1 Center for Molecular Innovation, The Feinstein Institute for Medical Research, 350 Community Drive, Manhasset, NY 11030 USA; 2 Division of Genetics and Cell Biology, Chromatin Dynamics Unit, San Raffaele University and San Raffaele Scientific Institute IRCCS, Via Olgettina 58, 20132 Milan, Italy; 3 Center for Biomedical Science, and Center for Bioelectronic Medicine, The Feinstein Institute for Medical Research, 350 Community Drive, Manhasset, NY 11030 USA

## Correction

After publication of this article (He et al., 2018), the corresponding authors recognised an error in Scheme 1, in particular to section “A. HMGB1/TLR4/MD-2 complex formation”. Above “Step 2: B box binding to MD-2”, the text incorrectly read: “Low affinity / extremely slow off”. In addition, some text was omitted below “TLR4/MD-2”. The correct version of Scheme 1 is included in this Correction article. The original article (He et al., 2018) has been corrected.

**Scheme 1:**
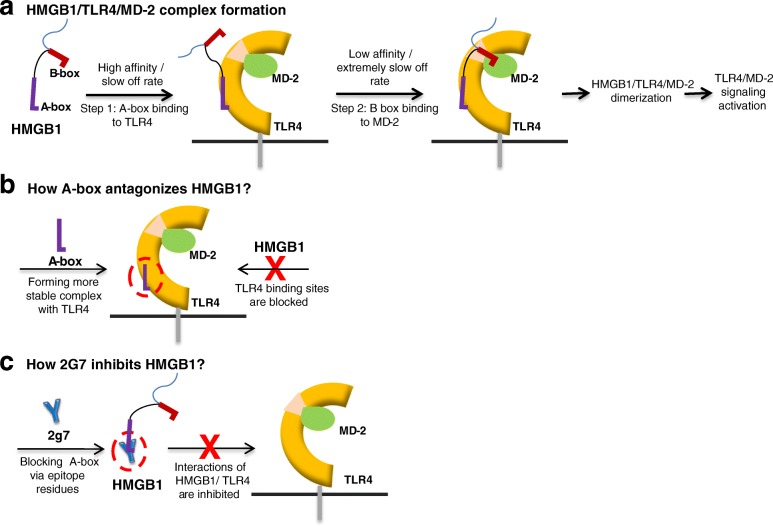
Proposed mechanism of HMGB1-TLR4 interaction and role of anti-HMGB1 antibody (2G7)
