# Supplementary material for: Exploring the biological functional mechanism of the HMGB1/TLR4/MD-2 complex by surface plasmon resonance
Source: Mol Med. 2018 May 10;24:21. doi: 10.1186/s10020-018-0023-8 (PMC6085627; doi:10.1186/s10020-018-0023-8)
Supplement: Supplementary file 1 — Table S1. Binding affinity of HMGB1 isoforms and segments. Table S2. Kinetics data of HMGB1 isoforms and segments. Figure S1. SPR analyses of GST-A-box and GST-B box binding to MD-2. Figure S2. SPR analyses of GST-A-box binding to TLR4/MD-2, TLR4. Figure S3. SPR analyses of GST-B-box binding to TLR4/MD-2, TLR4. Figure S4. SPR analyses of GST-A-box and A-box binding to TLR4/MD-2, TLR4. Figure S5. Ternary complex formation among TLR4, A-box and HMGB1. (DOCX 241 kb) [file 10020_2018_23_MOESM1_ESM.docx]

**Supplementary Data**

**Exploring the biological functional mechanism of the HMGB1/TLR4/MD-2 complex by Surface Plasmon Resonance**

**Running Head: Kinetics of HMGB1/TLR4/MD-2 complex formation or inhibition**

Mingzhu He^1^, Marco E. Bianchi^2^, Tom R Coleman^1^, Kevin J Tracey^3^, Yousef Al-Abed^1^

^1^Center for Molecular Innovation, The Feinstein Institute for Medical Research, 350 Community Drive, Manhasset, New York 11030, USA

^2^Chromatin Dynamics Unit, Division of Genetics and Cell Biology, San Raffaele University and San Raffaele Scientific Institute IRCCS, Via Olgettina 58, 20132 Milan, Italy

^3^Center for Biomedical Science, and Center for Bioelectronic Medicine, The Feinstein Institute for Medical Research, 350 Community Drive, Manhasset, New York, 11030, USA

**Corresponding authors:**

Dr. Mingzhu He & Dr. Yousef Al-Abed

Center for Molecular Innovation, The Feinstein Institute for Medical Research, 350 Community Drive,

Manhasset, NY, 11030, USA

Tel: +1-516-562-3406; Fax: +1-516-562-1022; Email: [mhe@northwell.edu](mailto:mhe@northwell.edu), [yalabed@northwell.edu](mailto:yalabed@northwell.edu)

**Contents**

Table S1. Binding affinity of HMGB1 isoforms and segments S2

Table S2. Kinetics data of HMGB1 isoforms and segments S3

Figure S1. SPR analyses of GST-A-box and GST-B box binding to MD-2 S4

Figure S2. SPR analyses of GST-A-box binding to TLR4/MD-2, TLR4 S4

Figure S3. SPR analyses of GST-B-box binding to TLR4/MD-2, TLR4 S4

Figure S4. SPR analyses of GST-A-box and A-box binding to TLR4/MD-2, TLR4 S5

Figure S5. Ternary complex formation among TLR4, A-box and HMGB1 S5

Table S1. Binding affinity of HMGB1 isoforms and segments


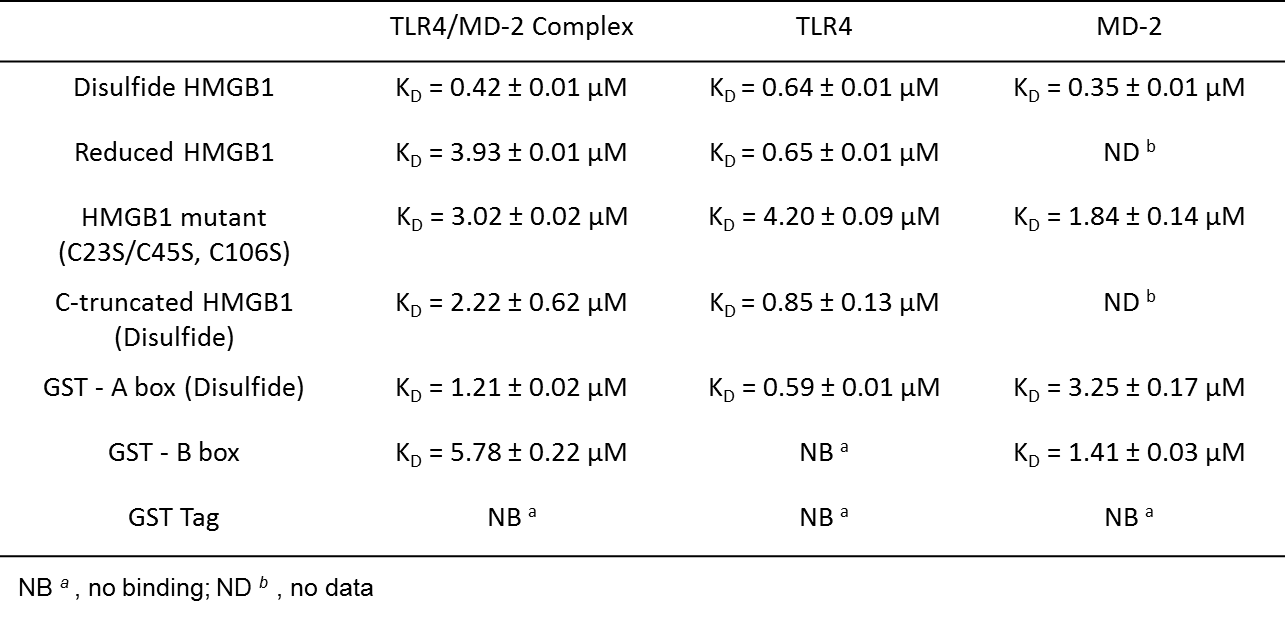


Table S2. Kinetics data of HMGB1 isoforms and segments


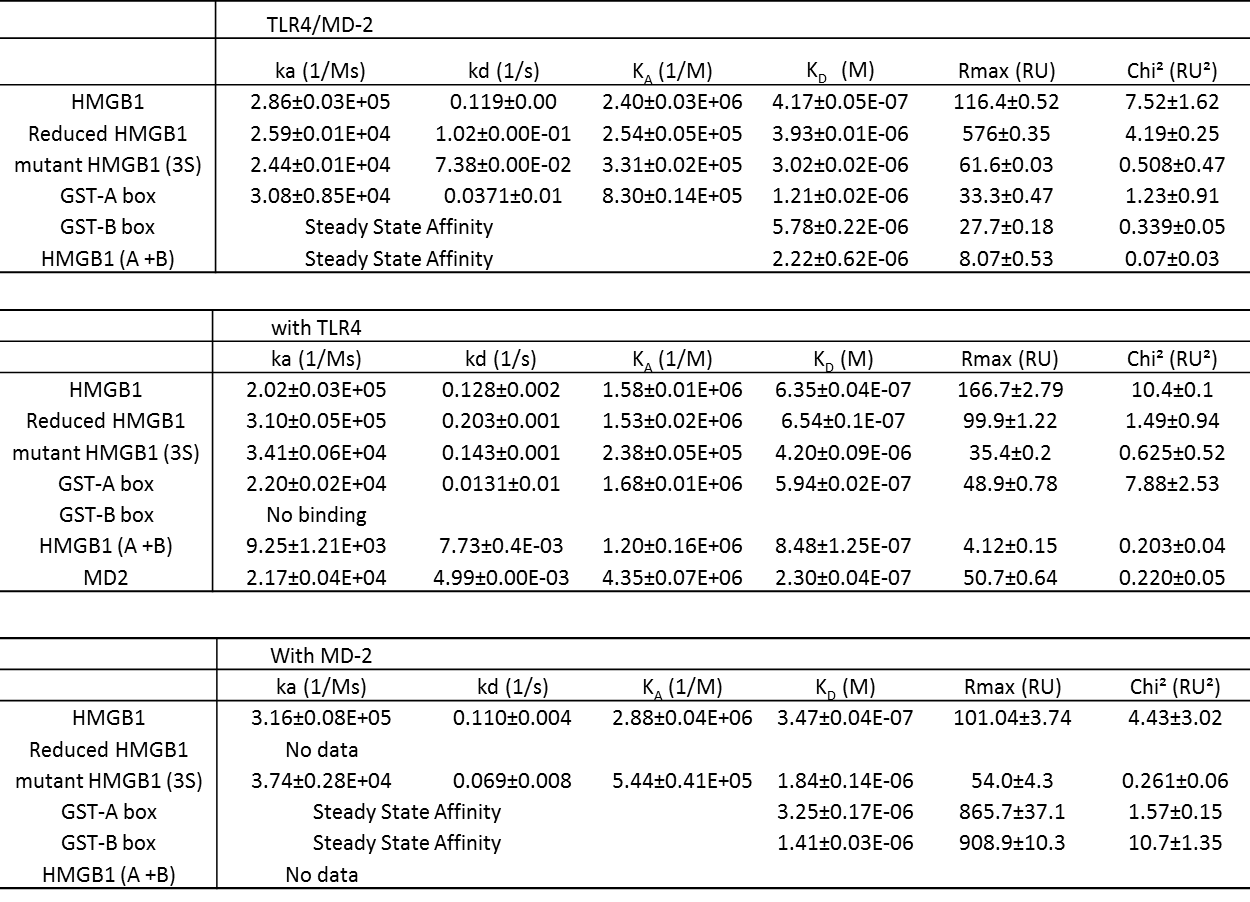


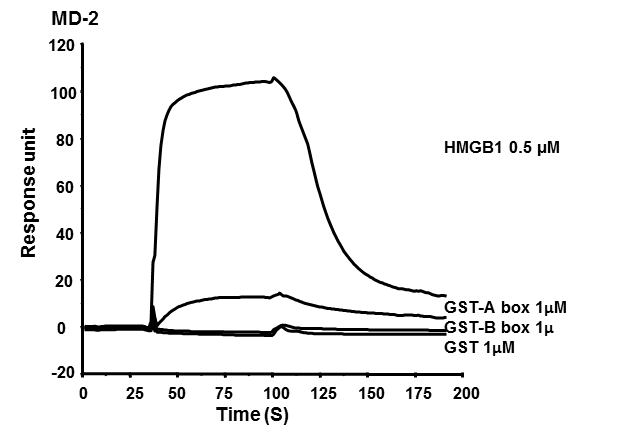


**Figure S1**. SPR analyses of GST-A-box and GST-B-box binding to MD-2

MD-2 was coated on the CM5 chip, GST-A-box (1µM), GST-B-box (1µM) and GST protein (1µM) were injected as analytes; GST protein has no binding to MD-2 and was used as negative control; HMGB1 (0.5 µM) was used as positive control. Data are representative of three repeats.


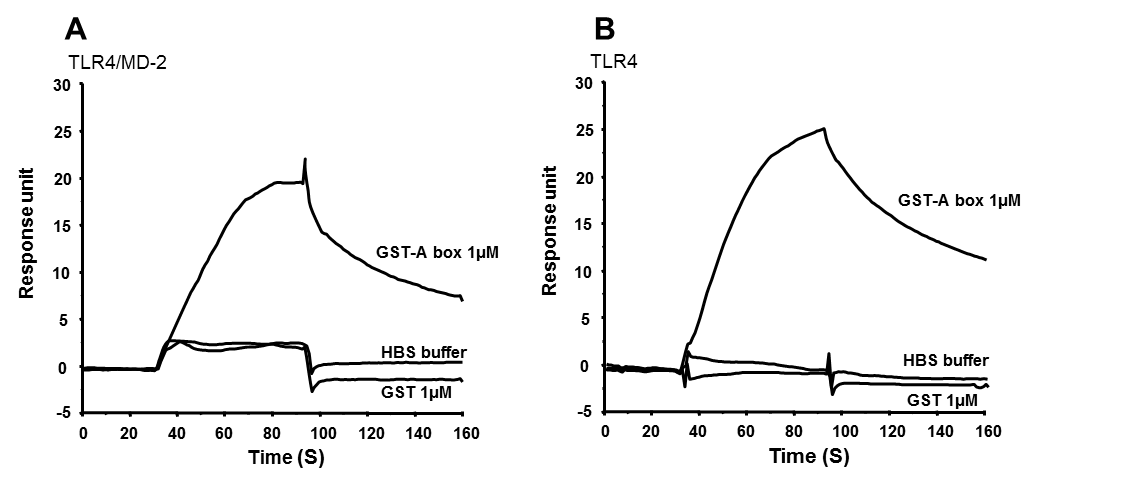


**Figure S2**. SPR analyses of GST-A-box binding to TLR4/MD-2, TLR4

(A) TLR4/MD-2 complex was coated on the CM5 chip, HBS buffer, GST-A-box (1µM) and GST protein (1µM) were injected as analytes. (B) TLR4 was coated on the CM5 chip, HBS buffer, GST-A-box (1µM) and GST protein (1µM) were injected as analytes. GST protein has no binding to TLR4/MD-2 or TLR4, and was used as negative control. Data are representative of three repeats.


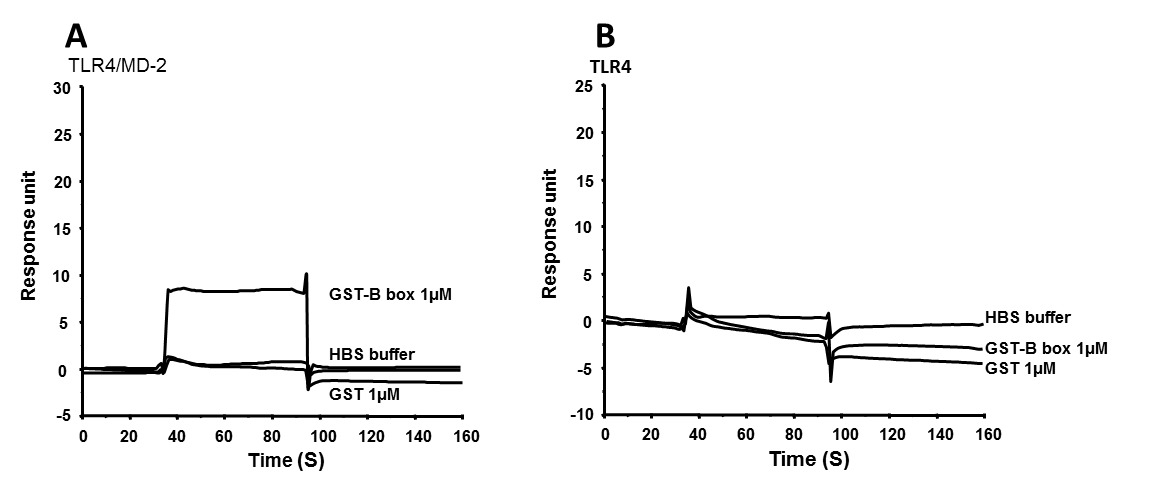


**Figure S3**. SPR analyses of GST-B-box binding to TLR4/MD-2, TLR4

(A) TLR4/MD-2 complex was coated on the CM5 chip, HBS buffer, GST-B-box (1µM) and GST protein (1µM) were injected as analytes. (B) TLR4 was coated on the CM5 chip, HBS buffer, GST-B-box (1µM) and GST protein (1µM) were injected as analytes. GST protein has no binding to TLR4/MD-2 or TLR4, and was used as negative control. Data are representative of three repeats.


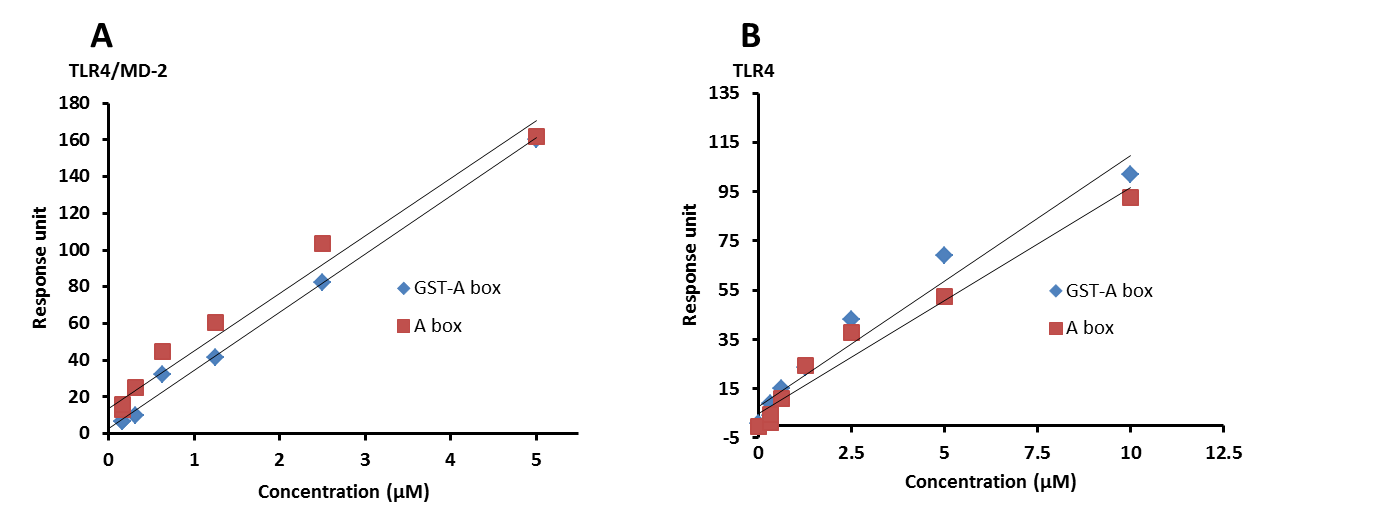


**Figure S4**. SPR analyses of GST-A-box and A-box binding to TLR4/MD-2, TLR4

(A) TLR4/MD-2 complex was coated on the CM5 chip; GST-A-box (0-5µM) and A-box protein (0-5µM) were injected as analytes. GST-A-box binds to complex with a K_D_ of 1.21 µM; A-box with a K_D_ of 1.55 µM. (B) TLR4 was coated on the CM5 chip; GST-A-box (0-10µM) and A-box protein (0-10µM) were injected as analytes. GST-A-box binds to TLR4 with a K_D_ of 0.59 µM; A-box with a K_D_ of 1.03 µM. Data are representative of three repeats.


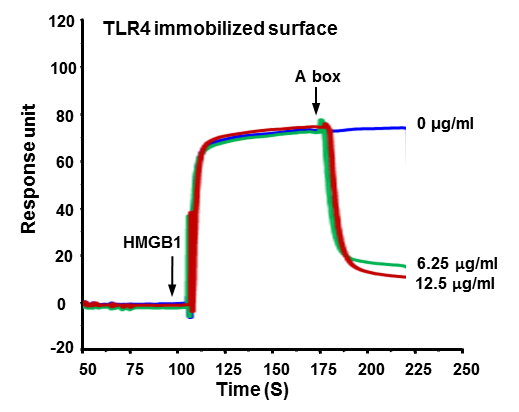


**Figure S5.** Ternary complex formation among TLR4, A-box and HMGB1

Biacore sensorgram to show molecular interactions, after injecting HMGB1 (0.5 µM), followed immediately by HMGB1 (0 µg/ml A-box) or A-box (6.25 and 12.5 µg/ml) onto a TLR4 sensor chip surface.
